# Mitochondrial Dysfunction in Propionic Acidemia: A Case‐Report and Review of the Literature

**DOI:** 10.1002/jmd2.70073

**Published:** 2026-02-04

**Authors:** Brandon K. Walther, Brittany M. Murray, Poornima Pandiyan, Randall Ray, Laura Yeoh, Amy Kritzer

**Affiliations:** ^1^ Division of Genetics and Genomics Boston Children's Hospital Boston Massachusetts USA; ^2^ Department of Pediatrics Harvard Medical School Boston Massachusetts USA; ^3^ Division of Medical Critical Care Boston Children's Hospital Boston Massachusetts USA

## Abstract

Propionic acidemia is an inborn error of metabolism involving an enzymatic defect of propionyl‐CoA carboxylase that results in the build‐up of toxic metabolites which can induce metabolic decompensation. Secondary mitochondrial dysfunction in propionic acidemia has been commonly recognized; however, its clinical presentation and management are not well represented in literature. Here, we present a case of profound hyperglycemia and lactic acidosis without hyperammonemia in a propionic acidemia patient, where medical management incorporated mitochondrial dysfunction via a brief reduction in glucose infusion rate. We review the literature on propionic acidemia and mitochondrial dysfunction in an effort to provide a tangible clinical case where considerations of mitochondrial dysfunction were made to guide further decision making in taking care of this patient population.

**Summary**

- We describe a rarely reported complication of propionic acidemia involving hyperglycemic crisis with lactic acidosis.
- Our interventions involved decision making centered around balancing the direct biochemical defects and energy metabolism deficiencies in organic acidemias.
- Trends of laboratory values before and after a reduction in glucose infusion rate, our main different intervention, are reported.
- We reflect on our decision making in a rarely reported but often fatal presentation of organic acidemias.

## 1 Introduction

Propionic acidemia (PA, OMIM #606054) is an inborn error of metabolism resulting from pathogenic variants in the *PCCA* or *PCCB* genes that encode the mitochondrial enzyme propionyl‐CoA carboxylase (PCC) [1]. PCC converts propionyl‐CoA to methylmalonyl‐CoA, which is further metabolized to succinyl‐CoA, a key citric acid cycle intermediate [2]. The clinical course is generally understood to encompass neurological and developmental differences, growth and feeding issues, cardiac disease, and hematologic abnormalities [3, 4, 5]. Secondary mitochondrial dysfunction, an additional component of the pathology [6], is hypothesized to be due to both a direct citric acid cycle deficiency and an accumulation of toxic metabolites [6, 7]. Despite this, it is unclear to what degree mitochondrial dysfunction contributes to the disease course, although it is implicated in cardiac, renal, and liver dysfunction [1].

Here, we present a patient admitted for acute metabolic decompensation requiring intensive care unit (ICU) disposition. Uniquely, while our patient displayed marked lactic acidemia and hyperglycemia, other components of metabolic decompensation were absent, with normal to modest ammonia levels incongruent with the severity of the other lab abnormalities. This presentation of PA with hyperglycemia and lactic acidosis is both rare and often fatal [8, 9, 10, 11, 12, 13, 14, 15, 16, 17]. In this case report, we review the literature on mitochondrial dysfunction in PA and the closely related methylmalonic acidemia (MMA, example entry: OMIM #251000), and describe the hospital course for our patient. We then discuss the role these biochemical defects may play in the acute management of PA, with a focus on the clinical differential for hyperglycemia in a PA patient, with the objective to suggest delineation of the various metabolic decompensatory scenarios.

## 2 Mitochondrial Disease in Propionic Acidemia and Review of the Literature

**FIGURE 1:**
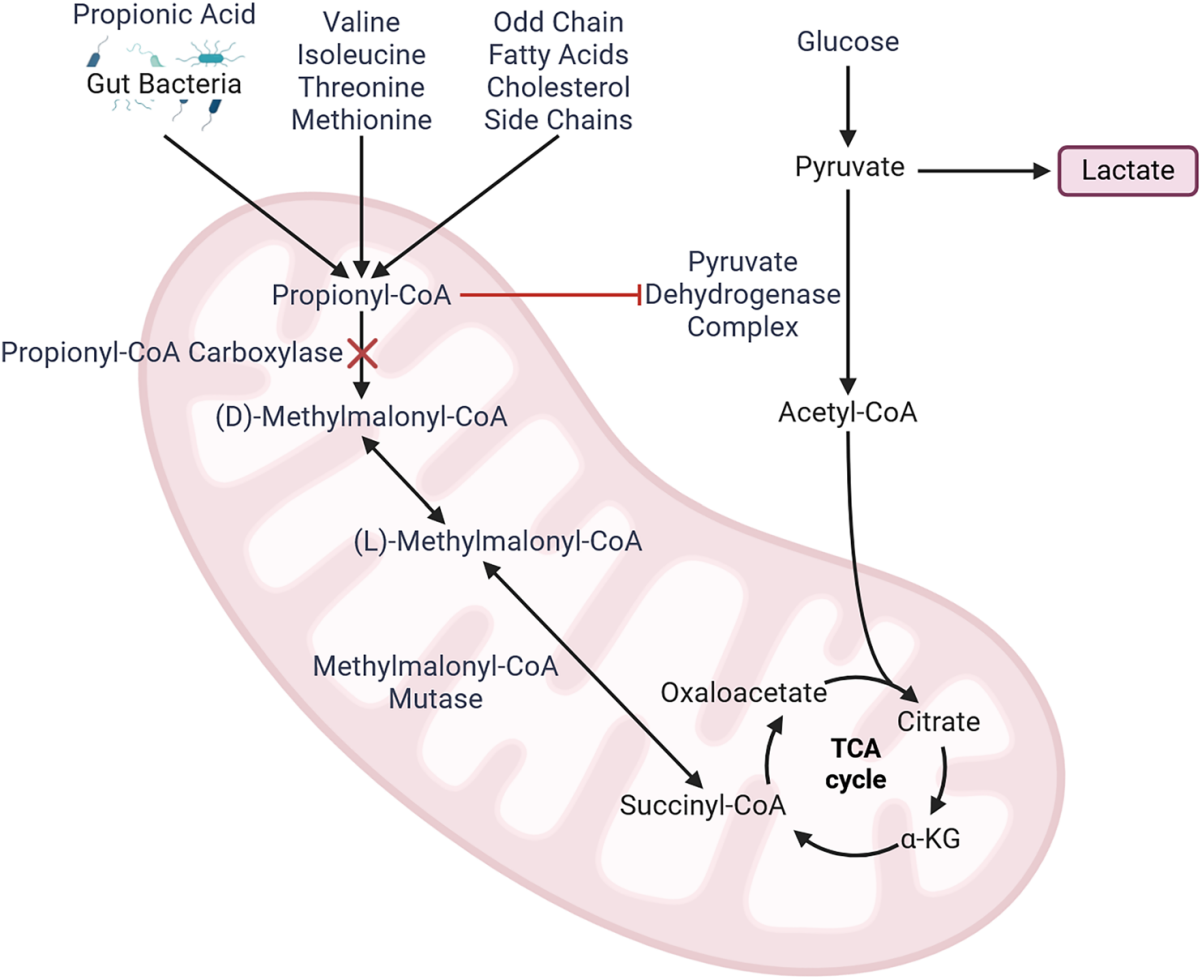
Biochemical Pathways in PA and MMA. Visually denoted defect reflects biochemical differences in PA. Created with BioRender.com.

Mitochondrial dysfunction in PA is a known component of the pathology [6] with multifactorial, incompletely understood mechanisms. There is a direct impact on energy metabolism downstream of the biochemical defects in PA (Figure 1), as PCC converts propionyl‐CoA to methylmalonyl‐CoA, which is then ultimately converted to succinyl‐CoA for use in the citric acid cycle [2, 18]. Deficiencies in any of the enzymes or cofactors responsible for converting methylmalonyl‐CoA to succinyl‐CoA result in methylmalonic acidemia, a closely related but distinct clinical entity [19]. In many cases, PA and MMA are treated similarly (and in this work, often we do the same as this is generally sound). However, there are a few biochemical and clinical distinctions to be noted, which we will discuss later in this section and throughout.

In addition to the direct downstream implications, mitochondrial disease in propionic acidemia is also thought to be due to toxic accumulation of propionyl‐CoA and its metabolites, including maleic acid, propionic acid, and 3‐hydroxypropionic acid [6, 20]. Several biochemical defects have been characterized. There is a direct inhibitory effect of propionyl‐CoA towards both pyruvate dehydrogenase and respiratory chain complex III^6^, and maleic acid has been shown to inhibit α‐ketoglutarate dehydrogenase complex [20]. The pathophysiologic response to these inhibitory effects is complex. There is an elevation of reactive oxygen species in models of propionic acidemia, although the magnitude of elevation appears to depend on the genetic variant and residual function [21]. Interestingly, mitochondrial DNA depletion has been observed in patients with PA and MMA [22]. Consequently, these defects may then induce protective physiologic adaptations to the oxidative and reductive stress through a variety of mechanisms, which are generally referred to as mitohormesis [23, 24]. The pathophysiology is overall characterized by a complex balance between increased oxidative and reductive stresses and compensatory changes. Here, it is important to add a key biochemical distinction between PA and MMA. Since the defects for MMA are in the following steps, MMA has accumulation of methylmalonyl‐CoA as a toxic substance which can then inhibit pyruvate carboxylase [25]. Inhibition of pyruvate carboxylase results in lactic acidosis. However, unlike pyruvate dehydrogenase deficiency where ketogenic diet is a mainstay of treatment [26], ketogenic diet is contraindicated in pyruvate carboxylase deficiency [27].

The clinical implications of the direct and indirect mitochondrial dysfunction are unclear, and much of the understanding of energy metabolism defects come from animal models [23]. There is literature support for respiratory chain complex dysfunction [23], and clinically, cardiomyopathy emerges as the most well supported pathology with observed mitochondrial dysfunction in patient samples [28, 29]. Across the multiorgan system involvement in PA and MMA, many of the clinical pathologies have been hypothesized to involve mitochondrial dysfunction, but of the many proposed mechanisms, cardiomyopathy, optic nerve involvement, and liver abnormalities have patient samples or treatments suggestive of a mitochondrial nature at this time [30].

The above picture makes it non‐trivial to assign mitochondrial dysfunction a definitive role in acute management [31, 32, 33], and acute management of PA focuses on reversing catabolism from a general perspective [31] with protein restriction, glucose infusion, and intralipid for caloric support. This is further complicated by a relative lack of data supporting specific mitochondrial medicines outside of a select few specific situations [34]. Nonetheless, based on the biochemical defects, both primary, from succinyl‐CoA depletion, and secondary, from inhibition of key enzymes in energy metabolism (such as pyruvate dehydrogenase), there is space to accommodate these paradigms in decision making, especially when metabolic decompensation is severe but does not clearly follow the usual patterns. Tangibly speaking, there are case reports in the literature discussing severe hyperglycemia and lactic acidosis mimicking diabetic ketoacidosis in PA and MMA [8, 9, 10, 11, 12, 13, 14, 15, 16, 17] which did not necessarily follow the usual clinical patterns observed in decompensation. Here, we discuss a similar presentation for a critically ill patient with known PA and reflect on our clinical decision making, with specific emphasis on interventions made or held based on known energy metabolism defects.

## 3 Patient Description

Our patient is a 6‐year‐old male diagnosed with PA biochemically on initial presentation. He initially presented on day of life 3 with severe metabolic acidosis and hyperammonemia to 582 μmol/L requiring emergency dialysis. An acylcarnitine profile obtained on day of life 3 showed elevated of C3 propionylcarnitine to 6.98 μmol/L, corroborated with his newborn screen returning out of range with a C3 level of 11.16 μmol/L. Urine organic acids showed elevations in 3‐hydroxypropionic acid, 2‐methylcitric acid, and 2‐methyl‐3‐hydroxybutyric acid, and small quantities of propionylglycine, consistent with a diagnosis of PA. This diagnosis was later confirmed after return of genetic testing showing biallelic pathogenic variants of the *PCCB* gene: c.990dup(p.Glu331*) and c.1173dup(p.Val392Cysfs*2).

His medical history includes hypotonia and significant developmental delay, G‐tube dependence, prior neonatal seizures previously on Keppra, and recurrent pancreatitis. By 15 months, he was rolling in both directions, sitting with support, and attempting to pull to stand. At 20 months, he was saying “dada” and “ma.” At 3 years of age, he was pulling to stand and cruising and babbling but did not have any clear vocabulary. At 6 years of age, he was crawling and cruising and was essentially non‐verbal. He has had numerous recurrent hospitalizations for metabolic crises. His last ophthalmology exam was at 2 years of age with no evidence of optic atrophy and normal visual acuity. Audiology testing at 3 years of age showed normal hearing bilaterally. His current renal function is normal and he has no structural heart disease, although he has had varying degrees of QTc prolongation. His home medications include carglumic acid, L‐carnitine, metronidazole, and a low protein modified diet and medical formula. The patient's weight for this hospitalization was measured at 18.6 kg. He does not have a baseline GDF15 level.

## 4 Hospital Course

Our patient presented to the emergency department with vomiting and fatigue. Upon arrival, he had tachycardia and low blood pressure. His exam on arrival was notable for sleepiness, but was otherwise unremarkable. He was admitted to the pediatric intermediate care unit for fluid rehydration, initially on D10W + ½NS at 1.5× maintenance rate (85 mL/h, glucose infusion rate/GIR of 7.6 mg/kg/h). Intralipids were initially held due to concern for pancreatitis. His home medications of carglumic acid at 1000 mg (approximately 50 mg/kg) 4 times daily and levocarnitine at 1200 mg (approximately 65 mg/kg, switched to IV on admission) three times daily were continued for the duration of the hospital course. During his stay in the intermediate care unit, he continued to have emesis and feeding intolerance, but progressed towards recovery over the next 2 days with slow advancement of his formula feeds. As his feeding advancement was progressing slowly, his fluids were adjusted to D12.5 W + ½NS at 1.5× maintenance (85 mL/h, GIR 9.5 mg/kg/min) to supplement his caloric intake overnight on hospital day 2–3. Then, on hospital day 3 at approximately 17:00, he had emesis and was unable to advance enteral feeds further, and labs were obtained.

**FIGURE 2:**
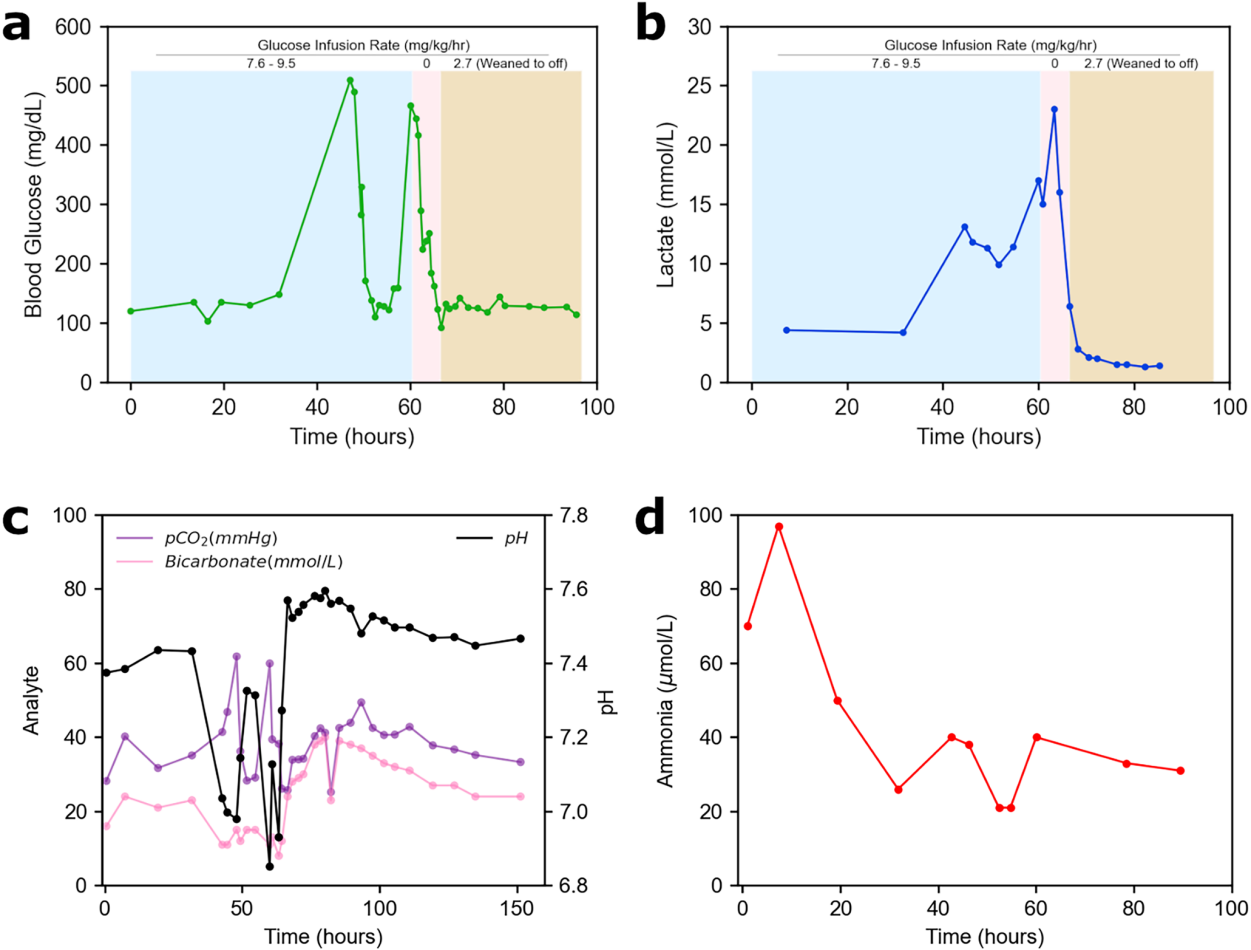
Various analytes monitored during the hospital course. (a) and (b) show the blood glucose and blood lactate level trends as they correlate with the glucose infusion rate (GIR) during the hospital course. The time of presentation to the ED is denoted as time 0, with each data point a single lab value obtained. (c) is a graph of the blood gases, which include both venous and arterial blood gases in the time course. Blood ammonia levels are shown in (d). Plotted bicarbonate is the calculated bicarbonate from the blood gas measurements. The shaded regions denote the time frames where the GIR was adjusted, with the red region showing the time frame when the GIR was 0 mg/kg/min.

The new labs showed that he was hyperglycemic to 558 mg/dL and his lactate levels rose to 13.8 mmol/L (Figure 2a,b), with an anion gap of 26 mmol/L. His venous blood gas showed a pH of 7.035, a bicarbonate of 11 mmol/L, and pCO_2_ levels peaking to 61.8 mmHg abruptly (Figure 2c). He was given 3 sodium bicarbonate boluses (1 mEq/kg/dose) during this first event, 2 normal saline boluses (20 mL/kg/dose), started on Intralipids (initially 1 g/kg/day then uptitrated to 2 g/kg/day), and started on empiric antibiotics of vancomycin and cefepime for sepsis evaluation. His fluids were adjusted at approximately 00:30 on hospital day 4 to ½NS at 0.75× maintenance (42 mL/h) and D12.5 W + ½Na Acetate at 0.75× maintenance (42 mL/h, GIR 4.7 mg/kg/min). An echocardiogram was obtained which was normal. Due to rising concern for pancreatitis, intralipids were discontinued after 4 h, although lipase obtained during this decompensation episode was 88 units/L (Figure S1).

While these interventions initially stabilized him overnight, the next morning of hospital day 4 at 11:00, he was hypothermic (35.5°C) with his VBG showing a pH of 6.85, a pCO_2_ level of 59.9 mmHg, a blood glucose of 466 mg/dL, and a lactic acid of 23 mmol/L (Figure 2a–c). His anion gap was 26 mmol/L. At this second exacerbation, his glucose infusion rate (GIR) was reduced to 0 mg/kg/min for 6 h (from hospital day 4 at 11:00 to 17:00), and fluids switched to a bicarbonate drip of 0.5 mEq/kg/h for approximately 1 h. An insulin drip was considered during this episode, but was not initiated due to concern that it would exacerbate lactic acidosis (see discussion). For caloric support, the plan was to initiate parenteral nutrition (PN) with intralipid starting at 1 g/kg/day (lipase obtained during this initial episode was 112 units/L). To bridge until the PN was prepared (at 17:00), fluids were adjusted from the bicarbonate drip to 160 mEq/L sodium acetate with 20 mEq/L potassium chloride at 1.5× maintenance (85 mL/h). These interventions markedly improved the hyperglycemia and lactic acidosis (Figure 2a,b). To control his GIR tightly at 17:00, the PN was constructed with a D5 base and 3/4:1/4 acetate: chloride balance to run at approximately 1× maintenance (60 mL/h, GIR 2.7 mg/kg/min), with 0.5× maintenance fluids left open on top for electrolyte and GIR fine tuning (24 mL/h). A mitochondrial cocktail was initiated of 100 mg thiamine daily, 100 mg coenzyme Q10 (ubiquinone) daily, and 75 mg/kg N‐acetylcysteine as a one‐time dose. Dialysis for lactic acidosis and altered mental status was discussed with the nephrology team, although was not initiated. He remained on PN for 48 h, and intralipid was uptitrated to 1.5 g/kg/day after 24 h (serially trended lipases peaked to 212 units/L at hospital day 4 at 15:05, but abruptly trended downwards after, see Figure S1).

Because of the second decompensation, we broadened the differential diagnosis and considered additional etiologies. Regarding the decision making around an insulin drip, additional lab work on hospital day 4 showed that his beta‐hydroxybutyrate (0.10 mmol/L) and hemoglobin A1C (4.5%) were unremarkable, and the endocrinology team confirmed that diabetic ketoacidosis was unlikely. Additionally, there were no signs of organ dysfunction (renal/hepatic) on serum studies during these episodes. Serial ammonia levels were also normal after the initial peak on presentation (Figure 2d).

Our patient continued on PN for 5 days following this episode while on a highly conservative reintroduction of home formula feeds. Trophic feeds were initiated on hospital day 6 with protein free formula, 48 h after the second decompensation episode to allow for gut rest. Intralipid was also discontinued on hospital day 6. On hospital day 8, he tolerated transition to his home feeding regimen at his home rate of 60 mL/h, and PN was fully discontinued. Other notable considerations, interventions, and work up also include that his infectious work‐up was negative. His triglyceride levels were all normal to borderline high (63–149 mg/dL). He also required BiPAP for respiratory support during the decompensation episodes (from hospital day 4 to 5), and volume resuscitation for hemodynamic support. A head CT obtained acutely was negative for stroke and edema, and EEG showed encephalopathy and no seizures. A brain MRI obtained after the episode showed no focal infarctions.

## 5 Discussion

Here, we report an unusual presentation of significant hyperglycemia and lactic acidosis in a patient with PA, and discuss the management considerations made. In the published literature thus far, this specific presentation is not uncommonly attributed to diabetic ketoacidosis prior to diagnosis and subsequent management shifts [8, 9, 10, 11, 12, 13, 14, 15, 16, 17]. Failure to respond to insulin is commonly reported during these cases [11, 12, 17], although not a universal finding [8, 10]. Given the rarity and severity of this clinical course, we add our patient's presentation and clinical course to the literature. In this report, we acknowledge that knowing our patient's diagnosis of PA allowed for shifting of the medical management rapidly given the known biochemical defects. In this section, we will discuss potential etiologies, our specific decision making, and reflect on what we would change or optimize if the clinical scenario were to arise again.

There are many etiologies for metabolic decompensation in patients with inborn errors of metabolism, and in PA this can range from a variety of infectious etiologies to more pathology specific findings like recurrent pancreatitis and cardiomyopathy [2]. Our patient had hypothermia, vital sign instability, hyperglycemia, and lactic acidosis. Hypothermia is a prognostic indicator in the critically ill patient, and while often associated with sepsis, may also be present in critically ill patients without [35]. Sepsis is a major, empirically treatable diagnosis given the hypothermia, lactic acidosis, and vital sign instability noted during the course. Additionally, though, hypothermia is a component of the PA clinical picture, often a presenting feature in the neonatal period [2]. This mechanism is hypothesized to be secondary to energy metabolism defects [36, 37, 38]. While this specific mechanism has not been directly studied and is inferred, generally speaking, research links hypothermia and temperature dysregulation to mitochondrial dysfunction [39, 40]. The presence of hyperglycemia also suggests specific pathologies, namely endocrine diagnoses. Diabetic ketoacidosis is an important consideration, especially as PA patients are at risk for exocrine dysfunction and insulin‐dependent diabetes [2]. With a normal ketone level and HgA1C, this was evaluated as unlikely in our case, but should be included in the clinical work up in presentations similar to this. Related to this diagnosis is pancreatitis. Recurrent pancreatitis is a common issue in patients with PA, and is also the major cause of diabetes in the population [2]. In our situation, we initially considered pancreatitis unlikely given the modest elevations in serially trended lipases. However, we do want to note 2 values cross threshold (taking 3 times the upper limit as normal to be 180 units/L, two recorded values of 190 units/L and 212 units/L, both on hospital day 3). Current guidelines emphasize that lipase levels do not correlate with clinical severity [41], and furthermore there are reports of, rarely, normal lipases in necrotizing pancreatitis [42, 43]. We did not obtain imaging of the pancreas during the hospital course due to low suspicion after the initial concern for pancreatitis was deemed low with his initial lipase levels. However, upon reflecting on our management, we would image the pancreas. This diagnosis has actionable management considerations and can present non‐specifically in a critically ill patient. Lastly, hyperglycemia and lactic acidosis are findings in severe stress states in the ICU [44, 45], which would serve to exacerbate many of the above etiologies, especially in patients with minimal physiologic reserve. As noted, our patient had normal ammonia levels, unusual for the degree of clinical decompensation, but nutritional supplementation/treatments during acute metabolic decompensation (lipids, carglumic acid, and carnitine) were continued as per guidelines [31, 32]. Lastly, we also want to acknowledge that delayed introduction of intralipid due to admission concern for pancreatitis may also have contributed.

We did not initiate insulin as we felt the risks for worsening acidosis were high due to the biochemical defects, which we will explore more fully. In all of the above cases, mitochondrial dysfunction is unlikely to be the primary driving factor, but it clearly can affect the clinical picture. Then, it follows to ask how the biochemical defects in PA and MMA would affect management. In most cases, this involves initiation of insulin during an acute decompensation. The primary decision point can be whether the acute decompensation is an insulin deficient state or not. While some cases are clear (e.g., diabetic ketoacidosis), practically, answering this question is non‐trivial. Furthermore, there is tension between the management decisions based on the underlying conceptual approach between insulin deficient states and biochemical mitochondrial inhibition. In an insulin deficient state (such as exocrine deficiency in pancreatitis), the treatment is, unsurprisingly, to provide insulin. This also provides several metabolic benefits beyond simply repleting a hormone deficiency. Insulin is strongly anabolic [46]. It drives glucose into cells [47] and inhibits protein breakdown [48] and ketogenesis [49]. All of these effects are beneficial during a catabolic crisis. In PA and MMA, however, this may present an issue, as the biochemical pathways for glucose processing are inhibited at multiple levels. TCA cycle inhibition from succinyl‐CoA depletion and secondary PDH inhibition raise the risk of insulin therapy then shunting glucose into lactate, worsening acidosis. This is directly called out in published guidelines for PA and MMA [32, 33]. Published case reports show varying responses to insulin infusion, while many recover with insulin treatment [10, 14, 15], others show persistent acidosis or required a reduction in glucose infusion rate [11, 16]. One critical confounding variable is that incorrectly diagnosing a PA or MMA metabolic decompensation as diabetic ketoacidosis is often fatal [13, 17]. Lastly, here it is important to highlight a key difference between PA and MMA. While both show PDH inhibition (and conceptually, we are borrowing glucose infusion reduction from PDH management [26]), MMA has increased levels of methylmalonyl‐CoA which inhibits pyruvate carboxylase [27]. Reducing glucose infusion in pyruvate carboxylase deficient states is contraindicated [27]. Thus, this proposed intervention likely carries higher risk in MMA compared to PA.

**FIGURE 3:**
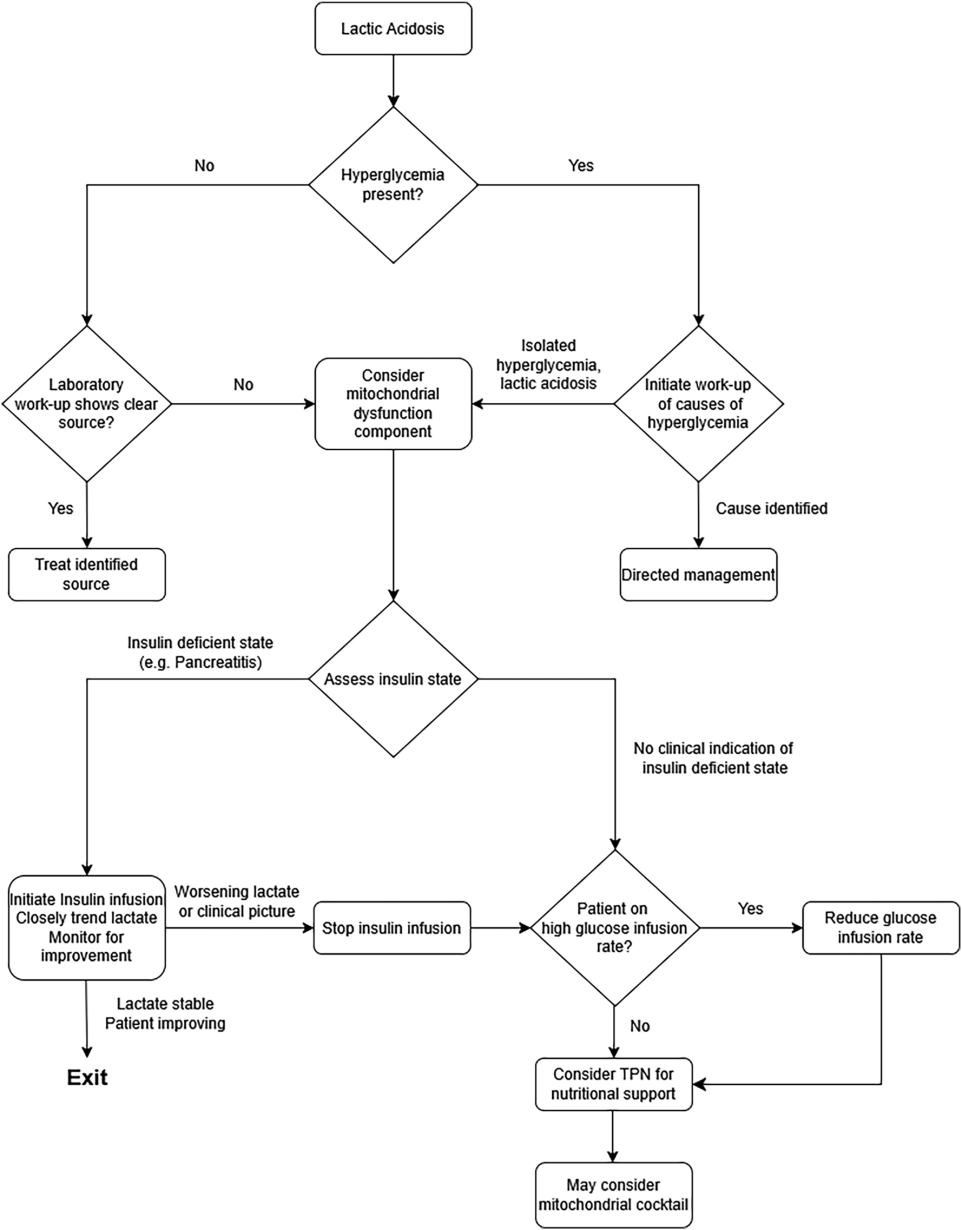
Decision making flowchart for isolated hyperglycemia and lactic acidosis in PA. Laboratory work‐up in this flowchart denotes directed investigation towards the given differential diagnosis for the clinical scenario, but commonly includes sepsis evaluation, pancreatitis evaluation, endocrine evaluation, and commonly obtained laboratory monitoring for PA in the acute setting.

Based on this, it is difficult to recommend a single intervention in all cases, especially as reducing glucose infusion is not a benign intervention and should only be done for short (on the order of hours) time periods to avoid inducing catabolism. Given the numerous benefits of insulin in metabolic decompensation, it is reasonable to trial insulin and closely monitor lactate levels, with a low threshold to stop this therapy and reduce glucose infusion for worsening acidosis. Alternatively, a short reduction in GIR would also be reasonable in the case where no clear insulin‐deficient state is present. We have put these suggestions into a decision chart (Figure 3) to organize the conceptual approaches. There is also a practical barrier in assessing mitochondrial dysfunction clinically during a decompensation episode, as measuring mitochondrial dysfunction in a definitive manner is difficult, especially during a decompensation episode. GDF15 and FGF21 are proposed markers [50, 51], but unfortunately they are also elevated in a variety of non‐mitochondrial disease cohorts [52], although FGF21 outperforms GDF15 in the latter case. Another marker, pyruvate, needs to be measured with lactate. Given the urgency of intervention, processing times for pyruvate may limit its utility in acute management. Nonetheless, these tests are available with other markers like glutathione, and during an acute decompensation, the information would be valuable even in retrospect. In future scenarios, we would send these markers to assess our clinical decision making.

Mitochondrial therapeutics exist, but given the general lack of literature support outside of specific cases [53], we cannot definitively recommend any specific intervention. Studies on therapies for mitochondrial dysfunction in PA and MMA are more scarce, although organ function improvement has been studied in MMA mouse models [54] (specifically: renal GFR and coenzyme Q10/Vitamin E treatment). Nonetheless, we used available literature to guide mitochondrial medical therapy when possible [34]. Our rationales are commonly seen. Thiamine, a cofactor of pyruvate dehydrogenase [55], is often employed in mitochondrial diseases under the premise that it will improve enzymatic function. Coenzyme Q10 and N‐acetylcysteine are antioxidants aimed at alleviating reactive oxygen species damage [34, 55]. However, these interventions have not strictly been shown to improve outcomes in the acute setting since control‐styled studies for metabolic patients are difficult for a variety of reasons. Commonly argued in favor of these interventions is that they are relatively benign and well‐tolerated, lowering the barrier to initiating these therapies in the ill patient [34]. At this time, we feel that it is most appropriate to leave this as a case‐by‐case decision, but would encourage their consideration. We also would not recommend any specific dosing for the same reasons as above and the variability in literature. In most cases, these are well tolerated medications and risk of initiation is low.

Lastly, dialysis was considered given the severe lactic acidosis, although it was not initiated as our patient responded to medical intervention. The benefit of this intervention in the critically ill patient with severe lactic acidosis remains unclear, as while it may offer a modality to infuse large amounts of base in the dialysate, its effect on outcomes is controversial [56, 57, 58]. Renal replacement strategies function as a bridge and only modestly reduce lactic acid levels since they do not improve the underlying lactic acid production [59]. Thus, the recommendation is generally to treat the cause. However, their use in mitochondrial disease has not been studied. It remains an open question whether the escalation to dialysis to correct physiologic parameters refractory to medical management in mitochondrial disease has an effect on outcomes, as in this case, that was the primary indication to consider this intervention.

To summarize, we present a case of severe hyperglycemia and lactic acidosis in a patient with PA. Our decision‐making included considerations of the primary and secondary biochemical defects in glucose metabolism, and we elected to briefly reduce glucose infusion rates, which normalized acidosis and blood glucose levels. Generally speaking, mitochondrial dysfunction will not be a primary driving factor for an acute decompensation; however, it can affect the response to a given intervention. We acknowledge that removing glucose infusion is not a benign management choice and instead provide a conceptual framework for initiating or withholding insulin infusions and when to discontinue these therapeutic interventions. At this time, we would not make any specific recommendations for mitochondrial therapy as there is a lack of evidence to substantiate consistent use, but given the relatively benign profile of these medications, they can be considered. Our hope is that this case and discussion will provide a roadmap for future similar presentations of PA and MMA, given the severity and poor outcomes.

## 6 Methods

Data were visualized using Matplotlib [60] and Seaborn [61] packages in Python 3.7.

## Funding

The authors have nothing to report.

## Conflicts of Interest

The authors declare no conflicts of interest.

## Supporting information


**Figure S1:** Lipase levels during the hospital course.

## Data Availability

The data that support the findings of this study are available on request from the corresponding author. The data are not publicly available due to privacy or ethical restrictions.

## References

[1] H. Marchukq , Y. Wang , Z. A. Ladd , X. Chen , and G.‐F. Zhang , “Pathophysiological Mechanisms of Complications Associated With Propionic Acidemia,” Pharmacology & Therapeutics 249 (2023): 108501. 37482098 10.1016/j.pharmthera.2023.108501 PMC10529999

[2] C. I. G. Aima , O. A. Shchelochkov , T. J. Serrano , and C. P. Venditti , “Propionic acidemia. GeneReviews[Internet],” 2024.

[3] L. Pena , J. Franks , K. A. Chapman , et al., “Natural History of Propionic Acidemia,” Molecular Genetics and Metabolism 105 (2012): 5–9. 21986446 10.1016/j.ymgme.2011.09.022

[4] S. C. Grünert , S. Müllerleile , L. De Silva , et al., “Propionic Acidemia: Clinical Course and Outcome in 55 Pediatric and Adolescent Patients,” Orphanet Journal of Rare Diseases 8 (2013): 1–9. 23305374 10.1186/1750-1172-8-6 PMC3568723

[5] J. Schreiber , K. A. Chapman , M. L. Summar , et al., “Neurologic Considerations in Propionic Acidemia,” Molecular Genetics and Metabolism 105 (2012): 10–15. 22078457 10.1016/j.ymgme.2011.10.003

[6] M. A. Schwab , S. W. Sauer , J. G. Okun , et al., “Secondary Mitochondrial Dysfunction in Propionic Aciduria: A Pathogenic Role for Endogenous Mitochondrial Toxins,” Biochemical Journal 398 (2006): 107–112. 16686602 10.1042/BJ20060221 PMC1525008

[7] D. M. Niyazov , S. G. Kahler , and R. E. Frye , “Primary Mitochondrial Disease and Secondary Mitochondrial Dysfunction: Importance of Distinction for Diagnosis and Treatment,” Molecular Syndromology 7 (2016): 122–137. 27587988 10.1159/000446586 PMC4988248

[8] I. M. Dweikat , E. N. Naser , A. I. Abu Libdeh , et al., “Propionic Acidemia Mimicking Diabetic Ketoacidosis,” Brain & Development 33 (2011): 428–431. 20634010 10.1016/j.braindev.2010.06.016

[9] W. Lehnert , A. Junker , H. Wehinger , et al., “Propionic Acidemia With Hypertrophic Pyloric Stenosis and Disturbances in Glucose Metabolism,” in Monatsschrift Kinderheilkunde: Organ Der Deutschen Gesellschaft für Kinderheilkunde (Springer, 1980), 720–723. 6110180

[10] R. Joshi and A. Phatarpekar , “Propionic Acidemia Presenting as Diabetic Ketoacidosis,” Indian Pediatrics 48 (2011): 164–165. 21378435

[11] L. Filippi , E. Gozzini , C. Cavicchi , et al., “Insulin‐Resistant Hyperglycaemia Complicating Neonatal Onset of Methylmalonic and Propionic Acidaemias,” Journal of Inherited Metabolic Disease 32 (2009): 179–186. 10.1007/s10545-009-1141-9 19588269

[12] R. L. Boeckx and J. M. Hicks , “Methylmalonic Acidemia With the Unusual Complication of Severe Hyperglycemia,” Clinical Chemistry 28 (1982): 1801–1803. 7046992

[13] M. Imen , B. Hanene , K. Ichraf , et al., “Methylmalonic Acidemia and Hyperglycemia: An Unusual Association,” Brain & Development 34 (2012): 113–114. 21802231 10.1016/j.braindev.2011.07.002

[14] P. Dejkhamron , K. Wejapikul , K. Unachak , P. Sawangareetrakul , P. Tanpaiboon , and D. Wattanasirichaigoon , “Isolated Methylmalonic Acidemia With Unusual Presentation Mimicking Diabetic Ketoacidosis,” Journal of Pediatric Endocrinology & Metabolism 29 (2016): 373–378. 26581066 10.1515/jpem-2015-0228

[15] A. Guven , N. Cebeci , A. Dursun , E. Aktekin , M. Baumgartner , and B. Fowler , “Methylmalonic Acidemia Mimicking Diabetic Ketoacidosis in an Infant,” Pediatric Diabetes 13 (2012): e22–e25. 21545677 10.1111/j.1399-5448.2011.00784.x

[16] N. Saini , A. Malhotra , S. Chhabra , and S. Chhabra , “Methylmalonic Acidemia Mimicking Diabetic Ketoacidosis and Septic Shock in Infants,” Indian Journal of Critical Care Medicine 19 (2015): 183. 25810618 10.4103/0972-5229.152776 PMC4366921

[17] F. Ciani , M. A. Donati , G. Tulli , et al., “Lethal Late Onset cblB Methylmalonic Aciduria,” Critical Care Medicine 28 (2000): 2119–2121. 10890676 10.1097/00003246-200006000-00078

[18] F. A. Hommes , J. R. G. Kuipers , J. D. Elema , J. F. Jansen , and J. H. P. Jonxis , “Propionicacidemia, a New Inborn Error of Metabolism,” Pediatric Research 2 (1968): 519–524. 5727920 10.1203/00006450-196811000-00010

[19] I. Manoli , J. L. Sloan , and C. P. Venditti , “Isolated Methylmalonic Acidemia,” 2016.

[20] A. C. Roginski , A. Wajner , C. Cecatto , et al., “Disturbance of Bioenergetics and Calcium Homeostasis Provoked by Metabolites Accumulating in Propionic Acidemia in Heart Mitochondria of Developing Rats,” Biochimica et Biophysica Acta (BBA) 1866 (2020): 165682. 10.1016/j.bbadis.2020.165682 31931102

[21] L. Gallego‐Villar , C. Pérez‐Cerdá , B. Pérez , et al., “Functional Characterization of Novel Genotypes and Cellular Oxidative Stress Studies in Propionic Acidemia,” Journal of Inherited Metabolic Disease 36 (2013): 731–740. 23053474 10.1007/s10545-012-9545-3

[22] S. Yano , L. Li , T. P. Le , et al., “Infantile Mitochondrial DNA Depletion Syndrome Associated With Methylmalonic Aciduria and 3‐Methylcrotonyl‐CoA and Propionyl‐CoA Carboxylase Deficiencies in Two Unrelated Patients: A New Phenotype of mtDNA Depletion Syndrome,” Journal of Inherited Metabolic Disease 26 (2003): 481–488. 14518828 10.1023/a:1025125427868

[23] N. Vardar Acar and R. K. Özgül , “The Role of Cellular Stress, Antioxidant System Response, Mitochondrial Function, and Metabolic Alterations in the Pathophysiology of Propionic Acidemia: A Systematic Review,” Journal of Cellular Physiology 240 (2025): e70072. 40763199 10.1002/jcp.70072

[24] Y.‐W. Cheng , J. Liu , and T. Finkel , “Mitohormesis,” Cell Metabolism 35 (2023): 1872–1886. 37939657 10.1016/j.cmet.2023.10.011 PMC10632604

[25] P. E. Head , J. L. Meier , and C. P. Venditti , “New Insights Into the Pathophysiology of Methylmalonic Acidemia,” Journal of Inherited Metabolic Disease 46 (2023): 436–449. 37078237 10.1002/jimd.12617 PMC10715492

[26] R. Ganetzky , E. M. McCormick , and M. J. Falk , “Primary Pyruvate Dehydrogenase Complex Deficiency Overview. GeneReviews[Internet],” 2021. 34138529

[27] M. L. D. Lasio , A. N. Lehman , A. Ahmad , and J. K. Bedoyan , “Pyruvate Carboxylase Deficiency. GeneReviews[Internet],” 2024.

[28] E. Alonso‐Barroso , B. Pérez , L. R. Desviat , and E. Richard , “Cardiomyocytes Derived From Induced Pluripotent Stem Cells as a Disease Model for Propionic Acidemia,” International Journal of Molecular Sciences 22 (2021): 1161. 33503868 10.3390/ijms22031161 PMC7865492

[29] M. Álvarez , P. Ruiz‐Sala , B. Pérez , L. R. Desviat , and E. Richard , “Dysregulated Cell Homeostasis and miRNAs in Human iPSC‐Derived Cardiomyocytes From a Propionic Acidemia Patient With Cardiomyopathy,” International Journal of Molecular Sciences 24 (2023): 2182. 36768524 10.3390/ijms24032182 PMC9916417

[30] H. A. Haijes , J. J. Jans , S. Y. Tas , N. M. Verhoeven‐Duif , and P. M. van Hasselt , “Pathophysiology of Propionic and Methylmalonic Acidemias. Part 1: Complications,” Journal of Inherited Metabolic Disease 42 (2019): 730–744. 31119747 10.1002/jimd.12129

[31] K. A. Chapman , A. Gropman , E. MacLeod , et al., “Acute Management of Propionic Acidemia,” Molecular Genetics and Metabolism 105 (2012): 16–25. 22000903 10.1016/j.ymgme.2011.09.026 PMC4133996

[32] M. R. Baumgartner , F. Hörster , C. Dionisi‐Vici , et al., “Proposed Guidelines for the Diagnosis and Management of Methylmalonic and Propionic Acidemia,” Orphanet Journal of Rare Diseases 9 (2014): 1–36. 25205257 10.1186/s13023-014-0130-8 PMC4180313

[33] P. Forny , F. Hörster , D. Ballhausen , et al., “Guidelines for the Diagnosis and Management of Methylmalonic Acidaemia and Propionic Acidaemia: First Revision,” Journal of Inherited Metabolic Disease 44 (2021): 566–592. 33595124 10.1002/jimd.12370 PMC8252715

[34] I. Barcelos , E. Shadiack , R. D. Ganetzky , and M. J. Falk , “Mitochondrial Medicine Therapies: Rationale, Evidence, and Dosing Guidelines,” Current Opinion in Pediatrics 32 (2020): 707–718. 33105273 10.1097/MOP.0000000000000954 PMC7774245

[35] D. Peres Bota , F. Lopes Ferreira , C. Mélot , and J. L. Vincent , “Body Temperature Alterations in the Critically Ill,” Intensive Care Medicine 30 (2004): 811–816. 15127194 10.1007/s00134-004-2166-z

[36] L. Gallego‐Villar , A. Rivera‐Barahona , C. Cuevas‐Martín , et al., “In Vivo Evidence of Mitochondrial Dysfunction and Altered Redox Homeostasis in a Genetic Mouse Model of Propionic Acidemia: Implications for the Pathophysiology of This Disorder,” Free Radical Biology & Medicine 96 (2016): 1–12. 27083476 10.1016/j.freeradbiomed.2016.04.007

[37] C. Morland , A. S. Frøland , M. N. Pettersen , et al., “Propionate Enters GABAergic Neurons, Inhibits GABA Transaminase, Causes GABA Accumulation and Lethargy in a Model of Propionic Acidemia,” Biochemical Journal 475 (2018): 749–758. 29339464 10.1042/BCJ20170814

[38] F. Feillet , O. A. Bodamer , M. A. Dixon , S. Sequeira , and J. V. Leonard , “Resting Energy Expenditure in Disorders of Propionate Metabolism,” Journal of Pediatrics 136 (2000): 659–663. 10802500 10.1067/mpd.2000.104290

[39] M. Rango , A. Arighi , C. Bonifati , R. del Bo , G. Comi , and N. Bresolin , “The Brain Is Hypothermic in Patients With Mitochondrial Diseases,” Journal of Cerebral Blood Flow and Metabolism 34 (2014): 915–920. 24619278 10.1038/jcbfm.2014.38 PMC4013774

[40] F. Cholley , P. Edery , D. Ricquier , S. Peudenier , A. Slama , and M. Tardieu , “Mitochondrial Respiratory Chain Deficiency Revealed by Hypothermia,” Neuropediatrics 32 (2001): 104–106. 11414641 10.1055/s-2001-13878

[41] S. Tenner , S. S. Vege , S. G. Sheth , et al., “American College of Gastroenterology Guidelines: Management of Acute Pancreatitis,” American Journal of Gastroenterology 119 (2024): 419–437. 38857482 10.14309/ajg.0000000000002645 PMC13221274

[42] S. DiGiacomo , R. Shakov , S. Khara , et al., “Acute Necrotizing Pancreatitis With Normal Amylase & Lipase: 615,” Official Journal of the American College of Gastroenterology| ACG 103 (2008): S238–S239.

[43] N. Rastogi and H. Sauthoff , “A Rare Entity: Necrotizing Pancreatitis With a Normal Serum Lipase: 1300,” Official Journal of the American College of Gastroenterology| ACG 112 (2017): S706.

[44] M.‐R. Losser , C. Damoisel , and D. Payen , “Bench‐To‐Bedside Review: Glucose and Stress Conditions in the Intensive Care Unit,” Critical Care 14 (2010): 1–12. 10.1186/cc9100 PMC2945096 20727232

[45] J. Epstein and M. J. Breslow , “The Stress Response of Critical Illness,” Critical Care Clinics 15 (1999): 17–33. 9929784 10.1016/s0749-0704(05)70037-3

[46] V. L. Tokarz , P. E. MacDonald , and A. Klip , “The Cell Biology of Systemic Insulin Function,” Journal of Cell Biology 217 (2018): 2273–2289. 29622564 10.1083/jcb.201802095 PMC6028526

[47] A. R. Saltiel and C. R. Kahn , “Insulin Signalling and the Regulation of Glucose and Lipid Metabolism,” Nature 414 (2001): 799–806. 11742412 10.1038/414799a

[48] H. Abdulla , K. Smith , P. J. Atherton , and I. Idris , “Role of Insulin in the Regulation of Human Skeletal Muscle Protein Synthesis and Breakdown: A Systematic Review and Meta‐Analysis,” Diabetologia 59 (2016): 44–55. 26404065 10.1007/s00125-015-3751-0

[49] A. Nadal , P. F. Marrero , and D. Haro , “Down‐Regulation of the Mitochondrial 3‐Hydroxy‐3‐Methylglutaryl‐CoA Synthase Gene by Insulin: The Role of the Forkhead Transcription Factor FKHRL1,” Biochemical Journal 366 (2002): 289–297. 12027802 10.1042/BJ20020598 PMC1222772

[50] J. M. Lehtonen , M. Auranen , N. Darin , et al., “Diagnostic Value of Serum Biomarkers FGF21 and GDF15 Compared to Muscle Sample in Mitochondrial Disease,” Journal of Inherited Metabolic Disease 44 (2021): 469–480. 32857451 10.1002/jimd.12307

[51] J. M. Lehtonen , S. Forsström , E. Bottani , et al., “FGF21 Is a Biomarker for Mitochondrial Translation and mtDNA Maintenance Disorders,” Neurology 87 (2016): 2290–2299. 27794108 10.1212/WNL.0000000000003374 PMC5270510

[52] L. G. Riley , M. Nafisinia , M. J. Menezes , et al., “FGF21 Outperforms GDF15 as a Diagnostic Biomarker of Mitochondrial Disease in Children,” Molecular Genetics and Metabolism 135 (2022): 63–71. 34991945 10.1016/j.ymgme.2021.12.001

[53] S. Parikh , A. Goldstein , M. K. Koenig , et al., “Diagnosis and Management of Mitochondrial Disease: A Consensus Statement From the Mitochondrial Medicine Society,” Genetics in Medicine 17 (2015): 689–701. 25503498 10.1038/gim.2014.177 PMC5000852

[54] I. Manoli , J. R. Sysol , L. Li , et al., “Targeting Proximal Tubule Mitochondrial Dysfunction Attenuates the Renal Disease of Methylmalonic Acidemia,” Proceedings of the National Academy of Sciences 110 (2013): 13552–13557. 10.1073/pnas.1302764110 PMC3746875 23898205

[55] A. J. Kuszak , M. G. Espey , M. J. Falk , et al., “Nutritional Interventions for Mitochondrial OXPHOS Deficiencies: Mechanisms and Model Systems,” Annual Review of Pathology: Mechanisms of Disease 13 (2018): 163–191. 10.1146/annurev-pathol-020117-043644 PMC5911915 29099651

[56] P. Y. Guo , L. J. Storsley , and S. N. Finkle , “Severe Lactic Acidosis Treated With Prolonged Hemodialysis: Recovery After Massive Overdoses of Metformin,” in Seminars in Dialysis, vol. 19 (Wiley, 2006). 10.1111/j.1525-139X.2006.00123.x 16423187

[57] J. A. Kraut and N. E. Madias , “Lactic Acidosis: Current Treatments and Future Directions,” American Journal of Kidney Diseases 68 (2016): 473–482. 27291485 10.1053/j.ajkd.2016.04.020

[58] S. Gaudry , P. M. Palevsky , and D. Dreyfuss , “Extracorporeal Kidney‐Replacement Therapy for Acute Kidney Injury,” New England Journal of Medicine 386 (2022): 964–975. 35263520 10.1056/NEJMra2104090

[59] J. Levraut , J. P. Ciebiera , P. Jambou , C. Ichai , Y. Labib , and D. Grimaud , “Effect of Continuous Venovenous Hemofiltration With Dialysis on Lactate Clearance in Critically Ill Patients,” Critical Care Medicine 25 (1997): 58–62. 8989177 10.1097/00003246-199701000-00013

[60] J. D. Hunter , “Matplotlib: A 2D Graphics Environment,” Computing in Science & Engineering 9 (2007): 90–95.

[61] M. L. Waskom , “Seaborn: Statistical Data Visualization,” Journal of Open Source Software 6 (2021): 3021.

