# Supplementary figures and images for: Mitochondrial Dysfunction in Propionic Acidemia: A Case‐Report and Review of the Literature
Source: JIMD Rep. 2026 Feb 4;67(2):e70073. doi: 10.1002/jmd2.70073 (PMC12872281; doi:10.1002/jmd2.70073)

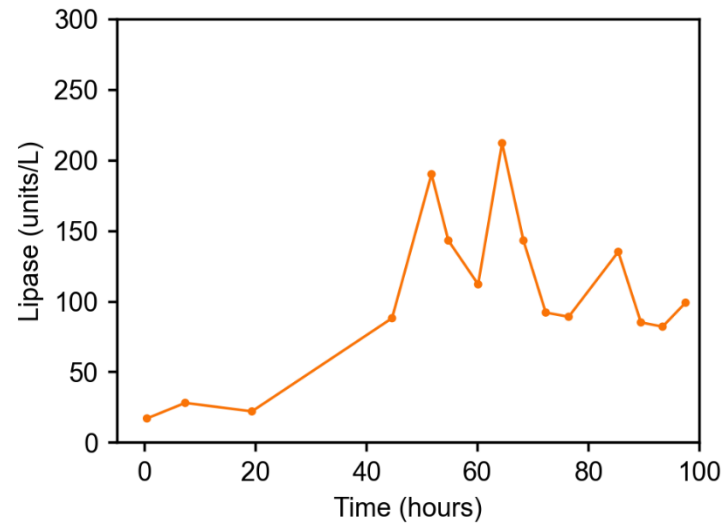

**Figure S1:** Lipase levels during the hospital course.

Supplement: Supplementary file 1 — Figure S1: Lipase levels during the hospital course. [file JMD2-67-e70073-s001.pdf]
